# Concomitant xanthogranulomatous pyelonephritis with renal abscess - an unusual cause of a right flank mass

**DOI:** 10.1590/S1677-5538.IBJU.2020.0641

**Published:** 2021-04-20

**Authors:** Valencia Long, Young Hwa Soon, Michelle Rui Ting Soo, Li Feng Tan

**Affiliations:** 1 National University Hospital System Alexandra Hospital Fast Program Singapore Fast Program, Alexandra Hospital, National University Hospital System, Singapore; 2 National University Hospital System Alexandra Hospital Division of Healthy Ageing Singapore Division of Healthy Ageing, National University Hospital System, Alexandra Hospital, Singapore

## INTRODUCTION

Xanthogranulomatous pyelonephritis (XGP) is an uncommon chronic granulomatous renal infection that can result in loss of renal function. Normal renal tissue is replaced by xanthogranulomatous material and serosanguinous fluid filled cysts infiltrated by lipid-laden macrophages (foam cells) (1). Women are more commonly affected than men (1).

We present a frail elderly lady with an unusual right sided flank mass, due to concomitant xanthogranulomatous pyelonephritis and renal abscess.

## CASE PRESENTATION

A frail, 89-year-old female presented with progressive functional decline and loss of appetite, after she had an unwitnessed fall 2 weeks ago.

**Figure 1:**
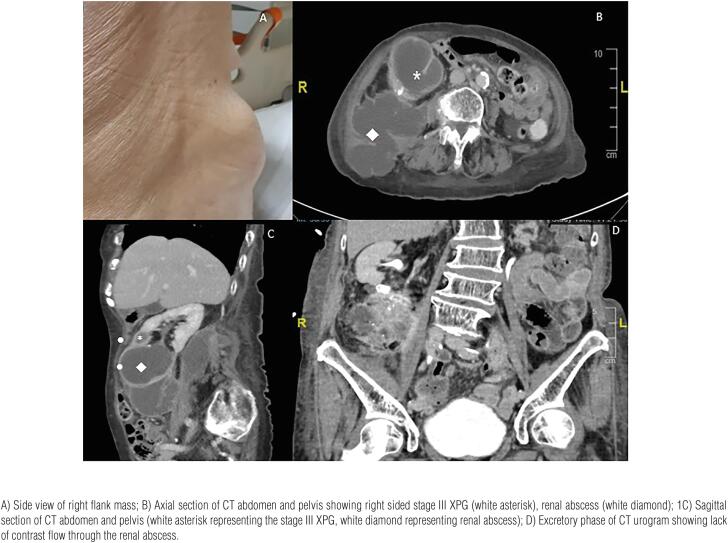
An 89 year-old female presenting with functional decline and a fall was discovered to have a right flank mass. She underwent CT abdomen and pelvis to evaluate the flank mass.

Past medical history was significant for well-controlled hypertension, osteoporosis, and cognitive impairment. She was febrile (temperature 38 degrees Celsius), blood pressure 110mmHg/80mmHg, pulse rate 85 beats per minute, saturating 100% on ambient air. Physical examination revealed a 5x5cm soft, right flank mass protruding from the posterolateral aspect of the right flank (Figure-1A).

Laboratory studies revealed leucocytosis (15.8x10^9^/L). She had pyuria on urine microscopy. Renal function was normal. She was treated empirically with intravenous co-amoxiclav for urinary tract infection.

A Computed Tomography (CT) of the abdomen and pelvis performed to evaluate the right flank mass revealed bilateral renal calculi and dilated calyces at the lower poles of both kidneys representing focal xanthogranulomatous pyelonephritis (XPG). On the left, the inflammatory changes extended into the perirenal fat (stage II XPG). On the right, these changes extended more extensively into the retroperitoneum and herniated through the posterior abdominal wall (stage III XPG), with concomitant renal abscess. Rim-enhancement of these cystic structures with surrounding mild fat stranding was seen, representing acute superimposed infection (Figure-1B, Axial View of the CT scan: white asterisk representing right sided stage III XPG, white diamond representing the renal abscess herniating through the posterior abdominal wall, Figure-1C, Sagittal View of the CT scan: white asterisk representing right sided stage III XPG, white diamond representing renal abscess).

She was referred to Urology and underwent an ultrasound-guided percutaneous drainage with two interlocking drains inserted for the right renal abscess located at the right inferior renal pole respectively. Fluid culture revealed Streptococcus anginosus. Cytology was compatible with acute suppurative inflammation. Urine culture revealed no growth. The drain was removed subsequently after a CT urogram confirmed near resolution of the collections. The excretory phase of the CT urogram confirmed that there was no passage of contrast through the renal abscess (Figure-1D). The patient remained stable and was eventually discharged with a one-month course of oral co-amoxiclav. There was no recurrence of the renal abscess.

## DISCUSSION

We present an uncommon cause of a right sided flank mass in a patient initially admitted for a fall, and then evaluated to have bilateral xanthogranulomatous pyelonephritis (XGP) with concomitant right sided renal abscess formation.

XGP can present acutely with urinary tract infection symptoms including dysuria, haematuria, fever. In chronic cases, non-specific symptoms of weight loss, malaise can arise. Causes of XGP include chronic renal obstruction, infection, abnormal lipid metabolism, lymphatic obstruction and renal ischemia. Abscess formation (as was observed in our patient), fistula formation (reno-cutaneous, reno-colonic) and profound sepsis are known complications (2).

Although the presenting symptoms of XGP may be similar to renal and perinephric abscesses, the imaging findings are distinct. Typical CT findings of XGP include renal enlargement and parenchymal inflammation. Multiple areas of low attenuation may be observed within the kidney, and these represent dilated renal calyces with pus-filled cavities replacing normal renal parenchyma. The characteristic thinning of the cortex associated with dilated calyces is also referred to as the “bear paw” sign (3). CT classification of XGP falls into 3 stages: stage I (nephric) is a localized disease confined to the renal parenchyma; stage II (perinephric) lesions involve perinephric fat; and stage III (paranephric) lesions extend beyond Gerota's fascia into the retroperitoneum (3).

The treatment for XGP involves the use of antimicrobials to achieve source control. Surgical treatment options include en-bloc nephrectomy, in which all the involved tissue is removed and any fistulas closed. In this patient with bilateral XGP, partial nephrectomy can be considered (4). Laparoscopic nephrectomy is an option, depending on the extent of the lesions and the experience of the managing urologist (5). Recurrent XGP may warrant partial or full nephrectomy.

The treatment for renal abscess arising as a complication of XGP usually involves antimicrobial therapy with percutaneous drainage especially when the abscess size is large (>5cm) (6, 7). When there is urological obstruction (such as obstructing calculus), the obstruction should be relieved. In cases where the abscess cannot be successfully treated with antibiotics and percutaneous drainage, surgical drainage may be warranted (8).

This patient was treated conservatively as she was frail and unfit for surgery. Percutaneous drainage was performed to decompress the renal abscess.

We emphasise that early awareness of the possible diagnosis and prompt investigations are important to avoid aggravation of the condition requiring more aggressive measures later on, especially in patients with possible predisposing factors.
